# Artificial intelligence and database for NGS-based diagnosis in rare disease

**DOI:** 10.3389/fgene.2023.1258083

**Published:** 2024-01-25

**Authors:** Yee Wen Choon, Yee Fan Choon, Nurul Athirah Nasarudin, Fatma Al Jasmi, Muhamad Akmal Remli, Mohammed Hassan Alkayali, Mohd Saberi Mohamad

**Affiliations:** ^1^ Institute for Artificial Intelligence and Big Data, Universiti Malaysia Kelantan, Kota Bharu, Kelantan, Malaysia; ^2^ Faculty of Data Science and Informatics, Universiti Malaysia Kelantan, Kota Bharu, Kelantan, Malaysia; ^3^ Faculty of Dentistry, Lincoln University College, Petaling Jaya, Selangor, Malaysia; ^4^ Health Data Science Lab, Department of Genetics and Genomics, College of Medicine and Health Sciences, United Arab Emirates University, Al Ain, United Arab Emirates; ^5^ School of Postgraduate Studies, United Arab Emirates University, Al Ain, United Arab Emirates

**Keywords:** rare disease, diagnosis, next-generation sequencing, artificial intelligence, machine learning, data science

## Abstract

Rare diseases (RDs) are rare complex genetic diseases affecting a conservative estimate of 300 million people worldwide. Recent Next-Generation Sequencing (NGS) studies are unraveling the underlying genetic heterogeneity of this group of diseases. NGS-based methods used in RDs studies have improved the diagnosis and management of RDs. Concomitantly, a suite of bioinformatics tools has been developed to sort through big data generated by NGS to understand RDs better. However, there are concerns regarding the lack of consistency among different methods, primarily linked to factors such as the lack of uniformity in input and output formats, the absence of a standardized measure for predictive accuracy, and the regularity of updates to the annotation database. Today, artificial intelligence (AI), particularly deep learning, is widely used in a variety of biological contexts, changing the healthcare system. AI has demonstrated promising capabilities in boosting variant calling precision, refining variant prediction, and enhancing the user-friendliness of electronic health record (EHR) systems in NGS-based diagnostics. This paper reviews the state of the art of AI in NGS-based genetics, and its future directions and challenges. It also compare several rare disease databases.

## 1 Introduction

Collectively, rare diseases (RDs) are a diverse group of heterogeneous diseases with approximately 7,000 distinct clinical entities. These diseases are commonly a result of genetic aberrations with early onset in children (Amberger et al., 2015; Wright et al., 2018; Tatiana and Tarailo-Graovac, 2019). Despite their rarity, RDs are emerging as a priority in global public health policy. An estimated 3.5%–5.9% of the world’s population (263–446 million persons) is burdened by RDs (Taruscio et al., 2010; Khosla and Valdez, 2018; Nguengang Wakap et al., 2020). RDs collectively affect a significant number of people worldwide. While each individual rare disease may impact only a small number of patients, when considered as a group, rare diseases have a substantial impact on public health. Furthermore, patients with RDs’ are challenged by: 1) the struggle to locate knowledgeable clinicians to diagnose and manage their conditions, resulting in delay-, under-or misdiagnosis, 2) costly disease-specific medications, 3) the struggle faced by clinicians to improve their competencies in managing RDs, which depends proportionately on the availability of the cases, and 4) difficulties in assembling cohorts of patients for clinical study, availability of drugs or devices, and a lack of funding to understand RDs better. Nevertheless, the emergence of various advocacy organizations and emerging genomics technologies have sped up the efforts to find cures and amelioration for this group of diseases (Elliott and Zurynski, 2015; Austin et al., 2018; Stoller, 2018; Liu et al., 2019a; Maroilley and Tarailo-Graovac, 2019; Baynam et al., 2020).

Rare diseases are inherently uncommon, there are typically severe constraints on available knowledge, research, medical expertise, and treatment options for each specific rare disease. Sharing clinical and genetic data on rare diseases can be challenging due to concerns about patient privacy and data security. Moreover, the rarity of the diseases causes the data available for each specific condition is limited. This scarcity of data makes it challenging to develop comprehensive databases and reference datasets. Rare diseases, by definition, have low prevalence. This means there is often a lack of reference data and comprehensive databases specific to these conditions. Consequently, it can be difficult to assess whether a specific genetic variant is pathogenic or benign. Variants of unknown clinical significance are common in rare diseases. These are genetic variations that are not clearly associated with disease or health. Interpreting VUS accurately is crucial for making informed clinical decisions and research advancements. As technology progresses, both public and scientific awareness has been increasing, and the accumulation, combination, and sharing of extensive data are set to greatly enhance our understanding of rare diseases (Hartley et al., 2020).

High throughput sequencing technologies are becoming an armamentarium for clinicians and researchers in modern medicine, especially in RDs (Grosse et al., 2010; Soon et al., 2013; Frésard and Montgomery, 2018; Amorim et al., 2019; Nguyen, 2019; Field, 2021). Next-generation sequencing (NGS) has been instrumental in discovering many underlying genetic aberrations of RDs. Such understanding has greatly improved the diagnosis and management of RDs (Jia and Shi, 2017; Fernandez-Marmiesse et al., 2018; Liu et al., 2019b; Rey et al., 2019; Vinkšel et al., 2021). Three NGS-based methods have exponentially identified disease-associated genes in the last 10 years, for example, the discoveries of novel genetic variants associated with age-related hearing loss (ARHL) (Girotto et al., 2019), Ménière’s disease (MD) (Gallego-Martinez et al., 2019) and severe congenital myasthenic syndrome with episodic apnea (CMS-EA) (Liu et al., 2019a) by targeted sequencing. It is becoming clear that genetic defects defining RDs are as heterogeneous as the disease (Liu et al., 2019b; Posey, 2019). Furthermore, the rapid accumulation of NGS-generated genomic data would challenge traditional sampling-based statistical methods’ ability to identify genetic pattern. Hence, more advanced computational techniques are in order, and artificial intelligence (AI) is fast becoming a method of choice (Cai et al., 2020). This paper summarizes the current uses of AI in NGS-based genetics and its future directions and challenges.

### 1.1 Targeted sequencing panels

Gene panels are used to anticipate the presence of pathogenic mutations associated with specific illnesses or disease groups by identifying specific genes or coding regions within genes (Rehm, 2013). Sequences can be sequenced to deeper levels than WES and WGS using targeted panels at a lower cost. In contrast to WES and WGS, detected variants are limited to a limited set of genes. And produce a minimal amount of data; as a result, the interpretation workload is reduced, and there is much less concern about incidental findings. However, panels need to be updated regularly in light of new knowledge and gene discoveries. WES and targeted panels have limitations in identifying structural variants, repetitive elements, and mitochondrial genetic variations (Miller et al., 2017).

### 1.2 Whole exome sequencing

The whole-exome sequence examines protein-coding regions of the genome, the regions of the genome that account for 1%–2% of the whole genome and are responsible for 95% of all diseases. It allows for identifying variants in genes that have not yet been linked to human genes (Rabbani, Tekin, and Mahdieh, 2014). An interpretation of WES can be provided with a preselected panel or a specific set of genes. Using bioinformatics panels, the laboratory can choose from gene lists associated with phenotypes of patients. It is also possible to compare the phenotype associated with these genes with the patient’s phenotype by looking at all rare and potentially damaging variants, (Yang et al., 2013). This approach enables the discovery of novel genes (novel gene association) by detecting previously undiscovered variants. Among WES’s limitations are the insufficient coverage of different regions, the limited ability to detect variations in repetitive elements, and variants in cases of somatic mosaicism. Further limitations include structural and deep intronic variants. Despite this, technology has continued to advance, enabling the method to cover exons more accurately and all disease-causing intronic variants, (Vinkšel et al., 2021).

### 1.3 Whole-genome sequencing

Human genomes can be largely mapped using whole-genome sequencing. The information obtained through genome sequencing promotes the discovery of new genes associated with diseases and gene modifiers that helps to answer complicated genetic inheritance questions (van El et al., 2013). Through this powerful tool, the genetic cause of many diseases can be discovered with only one test, which means it may become the most preferred genetic test in the future. WGS can detect several categories of genetic variation, including single-nucleotide variations (SNVs), insertions and deletions (indels), copy number variations (CNVs) and translocations (TLs) (Vinkšel et al., 2021). The potential benefits are unfortunately limited by the genome’s inaccessibility, cost, and complexity, as well as the current limitations of bioinformatics for interpreting non-coding genomic variants (Ormond et al., 2010). The WGS and WES methodologies have great potential for diagnosing rare diseases. They can analyze multiple genes in a single test while producing variants of unknown significance (VUS) and incidental findings. Hence, they pose additional challenges to clinicians and patients (Vinkšel et al., 2021).

## 2 NGS-based genetic diagnosis: challenges and opportunities

**FIGURE 1:**
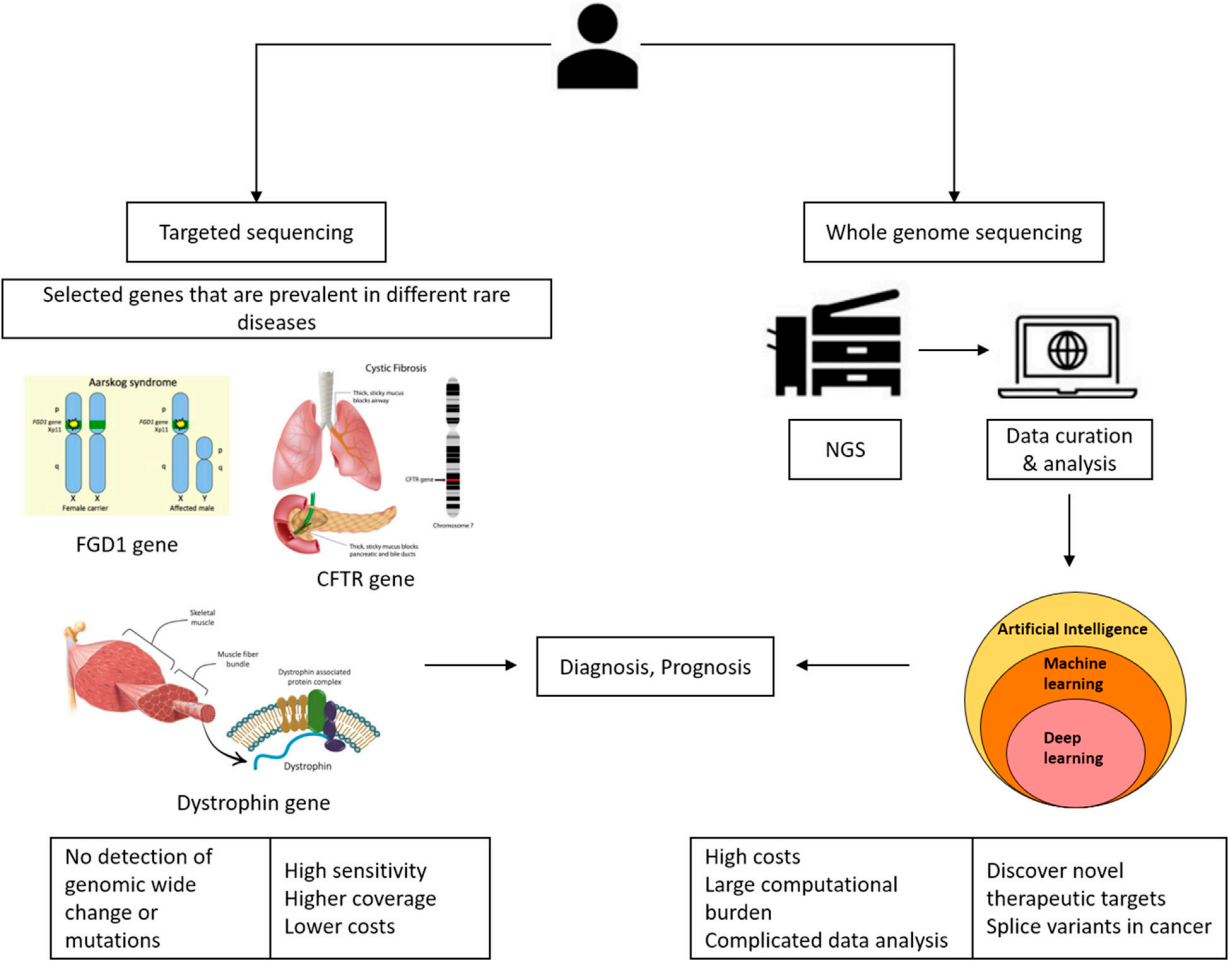
The use of sequencing in precision medicine.

NGS offers several advantages in the clinical setting for elucidating predictive or prognostic biomarkers. NGS has advanced significantly over the last decade, with considerable improvements in throughput, quality, cost, and sequencing time. State-of-the-art algorithms, along with their capacity to process vast and intricate datasets, present novel possibilities for precision medicine treatments. As depicted in Figure 1, sequencing plays a significant role in precision medicine. At present, targeted sequencing stands as the preferred approach for clinical applications due to its advantages, such as increased sensitivity, broader coverage, and cost-effectiveness. However, it has limitations, such as the inability to identify significant genomic rearrangements or potentially pathogenic mutations in non-targeted genes. The benefit of whole-genome sequencing is that it allows for mutations and alterations throughout the genome (Huang et al., 2019).

## 3 Artificial intelligence for enhancing NGS-based diagnosis

Ng et al. (2009) first demonstrated the use of NGS-based methods in RDs as a proof-of-concept that WES could identify candidate genes responsible for monogenic disorders like Freeman-Sheldon syndrome (FSS). Comparing their results to WGS, the group demonstrated high concordance, low false discovery rate, and equivalent sensitivity for cSNP detection of WES. In research related to rare diseases (RDs), WES has become the preferred method due to its cost-effectiveness and efficiency in collecting and analyzing genomic data compared to WGS and its superior ability to detect novel disease-causing genes than targeting sequencing. As the number of genes that NGS can sequence increases, more candidate genes will likely be found. One of the challenges faced by the increasing number of associated RDs genes is the bioinformatic tools currently used in the alignment, variant calling, and annotation of NGS-generated genomic data. The use of various software packages will yield distinct final interpretations, different statistical significance thresholds, and variant calling, ultimately resulting in a diverse final list of potential genes (Fernandez-Marmiesse et al., 2018).

**FIGURE 2:**
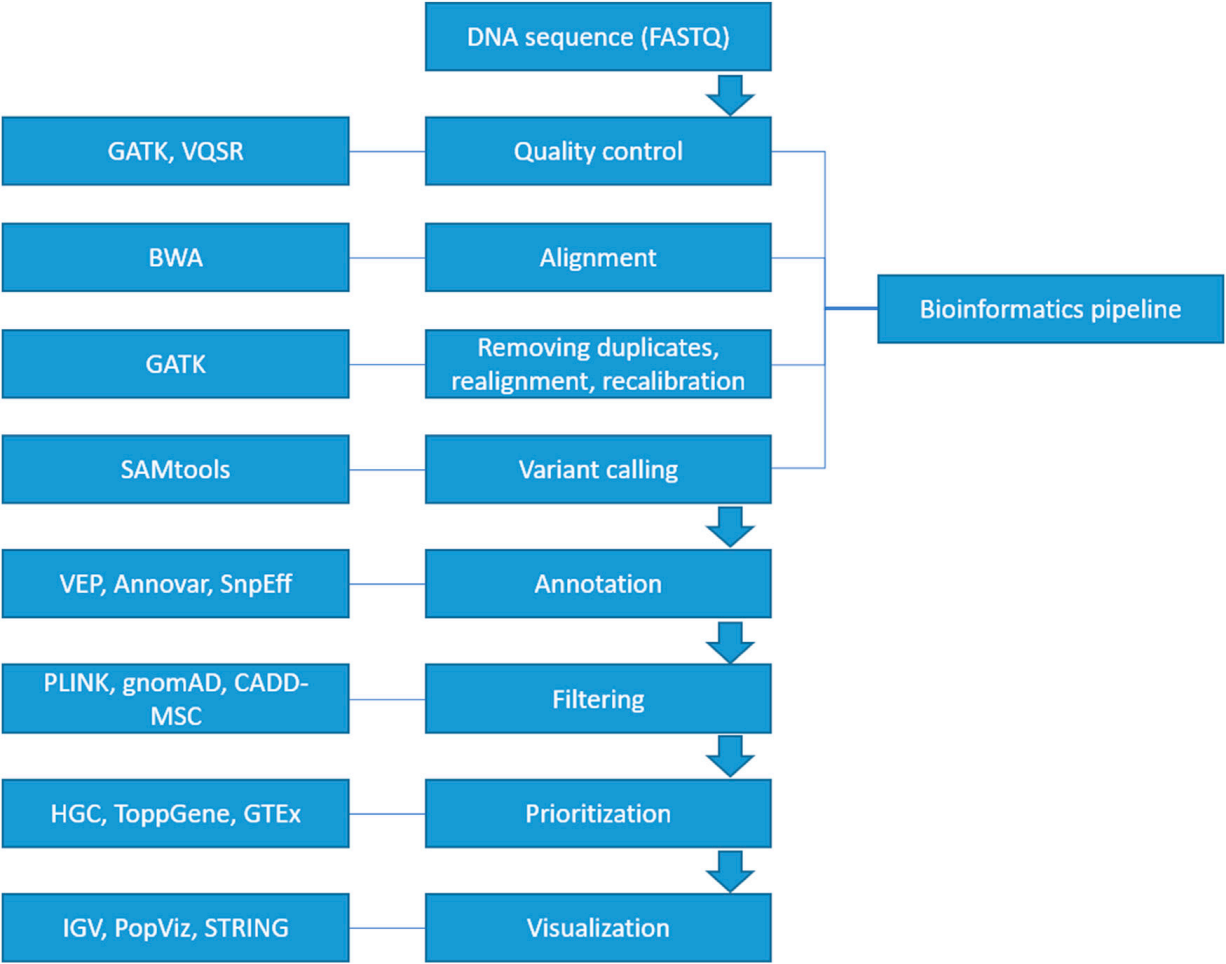
A framework of WES/WGS data analysis from individual patients with rare diseases. (GATK-Genome Analysis Toolkit, VQSR-Variant Quality Score Recalibration, BWA-Burrows-Wheeler Alignment, SAMtools-Sequence Alignment/Map tools, VEP-Ensembl Variant Effect Predictor, HGC-Hierarchical Graph-based Clustering, GTEx-Genotype Tissue Expression, IGV-Integrative Genomics Viewer).

**FIGURE 3:**
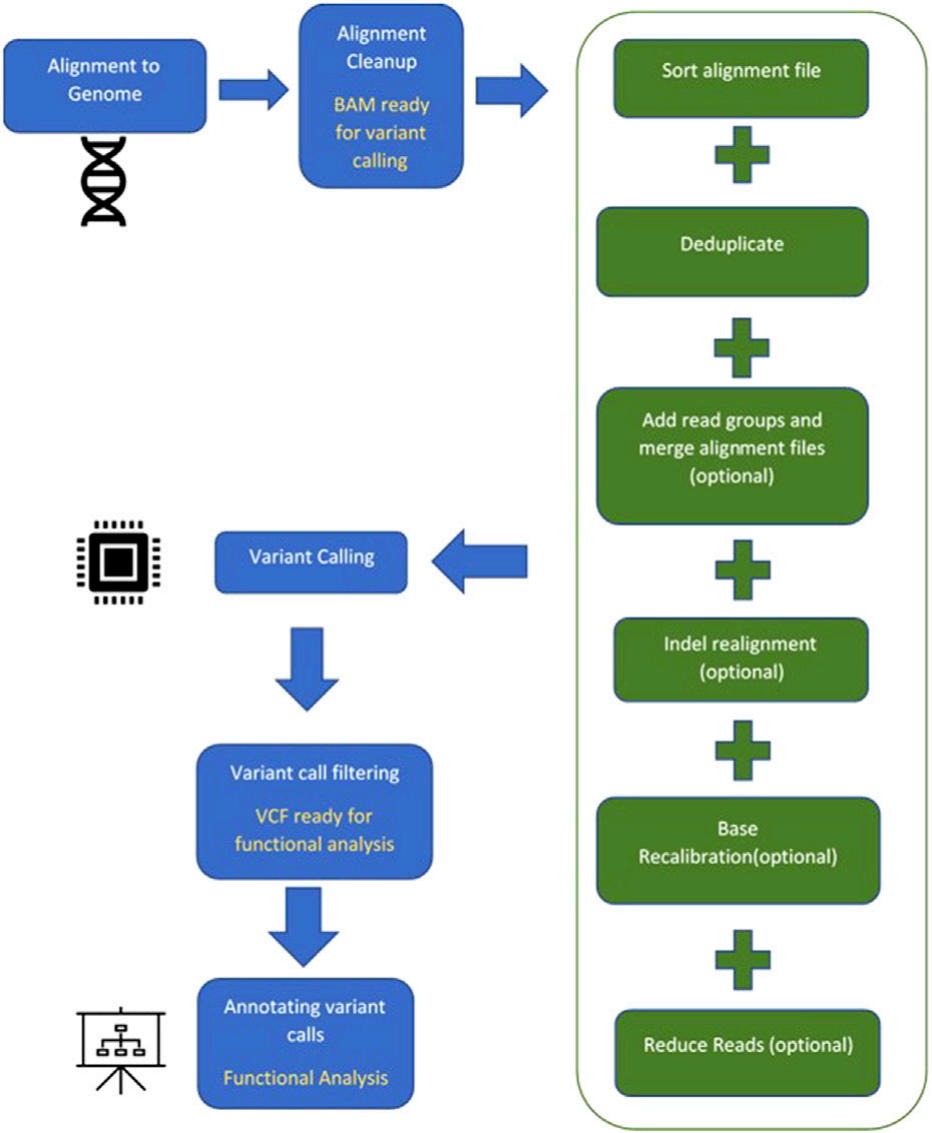
General workflow for NGS data analysis.

A suite of computational software is currently available for each step in identifying a diseasing-causing mutation in patients’ genomes. The use of bioinformatics in NGS-based genetic testing is essential. There are five key stages in the NGS bioinformatics pipeline that must be completed before suitable analyses can be performed. Figure 2 illustrates a framework of WES/WGS data analysis from individual patients with rare diseases, while Figure 3 illustrates the workflow for NGS data analysis. Recently, GIAB, together with the Global Alliance for Genomics and Health (GA4GH), has been actively creating benchmarking data to set a standard reference for adopting the most effective methods for NGS data analysis (Krusche et al., 2019; Zook et al., 2019).

Artificial intelligence (AI) has a worldwide and interdisciplinary influence. Today, AI, particularly deep learning, is widely used in various biological contexts, changing the healthcare system and other disciplines outside the scope of this paper. AI has significantly contributed to the analysis of next-generation sequencing (NGS) data. AI algorithms play a crucial role in automating and enhancing various facets of NGS data analysis, thereby increasing efficiency and precision. One prominent application of AI in NGS data analysis involves the alignment of sequences to a known reference genome. Alignment, which entails matching NGS-generated sequences to a reference genome, is a critical step in detecting genome variations and mutations. AI algorithms excel at streamlining this process by identifying the most suitable matching sequences and compensating for data errors or variations. AI also plays important role in the development of novel NGS data analysis tools and methodologies. For instance, AI can be harnessed to create algorithms capable of predicting the performance of various NGS assays or to discover innovative approaches to NGS data analysis that enhance accuracy and efficiency. The substantial role of AI in NGS data analysis lies in its capacity to automate and optimize numerous aspects of the process, ultimately rendering it more efficient and precise. The ability of AI algorithms to swiftly and accurately process vast quantities of data positions them as indispensable tools in the field of NGS data analysis.

Machine learning, a subfield of artificial intelligence (AI) and computer science, revolves around leveraging data and algorithms to emulate human learning and continuously enhance its accuracy. This technology holds the potential to revolutionize disease identification and treatment, significantly impacting clinical decision-making. As genomic data grows exponentially, conventional statistical sampling-based approaches face difficulties in identifying genetic patterns. This is where advanced algorithms like deep learning and AI become highly advantageous. By utilizing deep neural networks as an end-to-end method, complex feature patterns can be automatically extracted, and prediction models can be built with minimal manual feature engineering. Table 1 shows the advantages and disadvantages of clinical NGS analysis. Table 2 summarises the recent studies that use machine learing algorithms in NGS data analysis.

**TABLE 1:** Advantages and disadvantages of clinical NGS analysis.

Type analysis	Advantages	Disadvantages
Variant calling	- Essential for identifying genetic variants associated with diseases	- Can be complex, involving the analysis of large volumes of data generated by next-generation sequencing technologies
	- A valuable tool for studying population genetics	
Variant Filtering	- Allows researchers to focus on the most relevant and high-confidence variants	- Risk of Excluding True Positives
	- Can reduce the number of false-positive variants	- May inadvertently filter out variants of interest, leading to potential data loss
	- Making the subsequent steps of analysis faster and more manageable	
Variant Annotation and Prioritization	- Provides detailed information about the functional consequences of variants	- Variant annotation and prioritization can be complex
	- Helps researchers or clinicians focus on the most biologically relevant variants	- Require substantial computational resources
Phenotype-genotype association	- Can capture data from all over the genome, providing a comprehensive view of genetic variations	- Require large sample sizes for robust associations
	- Enables the detection of rare and novel variants	

**TABLE 2:** Summarises the recent studies that use machine learning algorithms in NGS data analysis.

Models	Algorithms	Notes	Refs
Variant Calling			
Deep Variant	Deep convolutional neural network (CNN)	The process of variant calling through short-read sequencing involves creating a representation of DNA alignments in the form of an image	Poplin et al. (2018)
Clairvoyante	Deep Convolutional neural network (CNN)	A CNN model with multitasking capabilities and adaptable for long sequencing data	Luo et al. (2018)
DeepNano	Deep recurrent neural network (RNN)	An advanced RNN for conduct base calling on MinION nanopore reads, yielding outcomes comparable to the performance achieved by Oxford Nanopore’s Nanonet base caller	Boža et al. (2017)
N/A	Logistic regression model	The creation of a deterministic machine-learning-based model aimed at distinguishing between two types of variant calls	van den Akker et al. (2018)
NeoMutate	Bayesian classifier, Bayesian model of admixture	Use of seven supervised machine learning algorithms, leveraging the advantages of various variant callers and integrating a unique collection of biological and sequence characteristics	Anzar et al. (2019)
	Heuristic methodology		
GATK HaplotypeCaller algorithm	N/A	A comprehensive pipeline designed to identify the optimal method for processing NGS data to accurately call variants for subsequent analyses with confidence	Pirooznia et al. (2014)
N/A	Multivariate linear regression	A machine learning method used to predict the quality scores of variant calls obtained from BWA + GATK	Cosgun and Oh (2020)
	Random forest regression		
	Neural network regression		
N/A	Random forests, adaptive boosting, k-nearest neighbors, naive Bayes, support vector machines	By combining multiple supervised machine learning techniques, the prediction of phenotype group associations significantly improves when relying on observed genotypes compared to using random permutations of the exomic sequences	Kringel et al. (2018)
Variant Filtering			
SNooPer	Random forest	A machine learning approach to call somatic variants in low-depth sequencing data	Spinella et al. (2016)
GARFIELD-NGS	N/A	A tool designed to distinguish between false and true variants in exome sequencing experiments	Ravasio et al. (2018)
Intelli-NGS	Deep neural network (DNN)	A tool based on deep neural networks that assists in minimizing false positive and false negative rates while maintaining high recall performance	Singh and Bhatia (2019)
DeepSVFilter	Convolutional neural network (CNN)	A deep learning-based approach for filtering structural variants in short genome sequencing data	Liu et al. (2021)
iEVA	N/A	A tool that amplifies informative features derived from NGS data and utilizes them in a filtering process employing a Machine Learning algorithm (ML)	Urtis et al. (2019)
DOMINO	linear discriminant analysis	A tool that evaluates the probability of a gene containing dominant alterations	Quinodoz et al. (2017)
Variant Annotation and Prioritization			
Skyhawk	Deep neural network (DNN)	An artificial neural networks that imitators the expert review process to detect clinically relevant genomic variants	Luo et al. (2018)
DANN	Deep neural network (DNN)	A DNN algorithm that surpasses state-of-the-art methods like support vector machine in predicting the deleterious annotation of genetic variants	Quang et al. (2015)
DeepSEA	Deep Convolutional neural network (CNN)	A deep CNN model utilized to predict the effects of noncoding variants directly from the sequence data and subsequently applied to forecast the functional impact of variants related to autism spectrum disorder	Zhou and Troyanskaya (2015)
eDiva	N/A	The framework integrates NGS data analysis, via functional annotation, and optimized causal variant prioritization	Bosio et al. (2019)
RENOVO	Random forest	An algorithm for reclassification of germline variants of unknown significance	Favalli et al. (2021)
Phenotype-genotype association			
DeepGestalt	Deep Convolutional neural network (CNN)	A sophisticated convolutional neural network model can distinguish rare diseases by analyzing patient face images and it can effectively discriminate different genetic subtypes	Gurovich et al. (2019)
DeepPVP	Deep neural network (DNN)	A Deep Neural Network model used for prioritizing variants by incorporating patients’ phenotype information	Boudellioua et al. (2019)
Xrare	N/A	A method to prioritize causative gene variants in the diagnosis of rare diseases	Li et al. (2019)
SQUIRLS	Random forest	An algorithm in classifying splice variants	Danis et al. (2021)

N/A represents that the information is not reported in the paper.

### 3.1 Variant calling

The task of detecting variants from sequencing data is referred to as variant calling. Despite the existence of several variant calling algorithms, many of them still require improvement, especially in clinical settings. Machine learning-based algorithms offer an alternative approach for variant calling.

### 3.2 Variant prediction

The clinical implementation of NGS-based diagnosis faces a hurdle in distinguishing pathogenic mutations from benign genetic variations. Despite the creation of various variation effect prediction tools to bridge this gap, it still constitutes a limiting factor that necessitates further validation in the decision-making process (Xu et al., 2019).

### 3.3 EHR

Connecting genetic testing to EHR systems is essential to integrating genomics into clinical practice (Abul-Husn and Kenny, 2019). Meanwhile, the electronic health record (EHR) system has served as a centralized platform for integrating diverse digital health data, leading to improved clinical decision-making and precision medicine. The difficulty lies in integrating data profiles of different complexities within the EHR system to enhance clinical diagnosis. AI advancements offer a potential solution to this challenge.

### 3.4 Phenotypes and genetic testing association

The main objective of a genetic association study is to investigate whether a particular sequence, such as a chromosomal region, haplotype, gene, or allele, plays a role in determining specific traits, metabolic pathways, or diseases. Deep learning has been widely used to improve diagnosis performance in medical image diagnostic systems, outperforming radiologists and pathologists (Yu et al., 2018). For example, DeepGestalt proposed by Gurovich et al. (2019) included over 17,000 pictures for over 200 rare diseases and reached 91% accuracy.

## 4 Databases for rare diseases

AI and NGS complement each other exceptionally well since AI thrives on extensive data while NGS generates vast amounts of data. Alongside the massive NGS data, other diagnosis-related testing data is also being produced, presenting the challenge of adequate data storage. To securely manage this data, a sophisticated informatics infrastructure is necessary. Measures have been taken to ensure that cloud-based services adhere to health privacy regulations, allowing for the secure storage of NGS data and the establishment of standardized data privacy practices among various stakeholders (Langmead and Nellore, 2018).

Although AI holds promise for improving clinical diagnosis in rare diseases, its effectiveness can be hindered by the intricate and diverse profiles of clinical data. Constructing an AI model for diagnosing rare diseases requires a substantial training dataset comprising patients with documented clinical outcomes. This paper reviews a few currently available databases for rare disease diagnosis. Table 3 summarises the available databases for rare diseases. Table 4 shows comparison between available databases for rare diseases.

**TABLE 3:** Summarises the available databases.

Database	Description	URL	Reference
NORD	An organization that support individuals affected by rare diseases and the entities that offer them assistance	https://rarediseases.org/for-patients-and-families/information-resources/rare-disease-information/	NORD (2021)
GARD	Offers the general public reliable, up-to-date, and user-friendly information about rare or genetic diseases	https://rarediseases.info.nih.gov/diseases	GARD (2021)
Orphanet	The goal is to gather limited information about rare diseases in order to improve the diagnosis, care, and treatment of patients afflicted by these conditions	https://www.orpha.net/consor/cgi-bin/Disease.php?lng=EN	Orphanet (2021)
OMIM	An ever-evolving repository of human genes, genetic disorders, and traits, with investigating the relationship between genes and phenotypes	https://www.omim.org/	Amberger et al. (2015)
LORIS MyeliNeuroGene	Natural history studies and clinical trial readiness	https://myelineurogene-stg.loris.ca/	Spahr et al. (2021)

Note: N/A represents unavailable information.

**TABLE 4:** Comparison of available databases for rare disease.

Database	Services	Advantage	Disadvantage
NORD	Offers detailed information on rare diseases, patient advocacy, support groups, and patient assistance programs	- Highly patient-centered and offers extensive support, advocacy, and information for individuals and families affected by rare diseases	- The focus is mainly on rare diseases, and it may not be as comprehensive in terms of genetic and molecular information
GARD	Offers information specialists for personalized assistance, educational materials, and government-funded resources	- Freely accessible to the public	- While it provides extensive information, it may not have the same level of patient support and advocacy as NORD
		- Comprehensive information on genetic and rare diseases, including disease descriptions, research, clinical trials, and expert guidance	
Orphanet	Offers information for both healthcare professionals and the general public	- Provides information on rare diseases, orphan drugs, expert centers, and research projects	- Primary focus is on Europe, and some information may be less relevant for non-European users
OMIM	Offers extensive genetic and molecular information, including genetic mutations and associated clinical features	- Specializes in the genetic and molecular basis of human diseases and disorders	- Primarily focuses on monogenic disorders and may not provide comprehensive information on complex genetic traits or disorders influenced by multiple genes and environmental factors
		- Freely accessible to the public	
LORIS MyeliNeuroGene	Offers information on rare neurological conditions, clinical trials, and genetic research	- Focuses on rare neurological diseases and disorders, particularly those affecting the central nervous system	- Limited to rare neurological diseases, so it may not be relevant for individuals seeking information on other types of rare diseases
			- Funding sources may not be as transparent as those of larger, more established resources

### 4.1 National organization for rare disorders (NORD) rare disease database

Since its inception in the early 1980s, coinciding with the implementation of the Orphan Drug Act, the National Organization for Rare Disorders (NORD) has been functioning as a support and advocacy organization for those individuals impacted by rare diseases. The database subscribers are granted entry to extensive monographs containing detailed information about the causes, symptoms, standard and investigational treatments, as well as support organizations related to various rare diseases. The level of detail offered in these monographs exceeds that of other resources, making it highly valued by patients and their families.

The Rare Diseases Database presently comprises data on over 1,200 diseases, Organized in alphabetical sequence or capable of being searched by disease name or synonym. It is important to note that NORD clarifies this database is not exhaustive, given that there are nearly 7,000 acknowledged rare diseases. As a non-profit advocacy organization, NORD’s resources for this informational database are limited, and it chooses to rely on volunteer specialists to contribute material.

### 4.2 NIH genetic and rare diseases information center (GARD)

The NORD Rare Diseases Database has a limited scope, so the website provides links to additional resources, especially the NIH Genetic and Rare Diseases (GARD) Information Center. The main objective of GARD is to provide up-to-date, precise, and easily understandable information regarding rare or genetic diseases in both English and Spanish. The GARD Information Center database contains approximately 6,700 specific diseases, and the data is generated by “information experts” with genetics degrees, according to the GARD Operations Manager (Hogan Smith, 2017).

Some information on the listed diseases is sourced from external databases like Orphanet, a European rare disease database. While GARD covers more rare diseases than the NORD Database, some entries require additional information.

GARD also allows users to ask questions to a GARD information professional. The responses are akin to a librarian’s helpful response to consumer health information queries, often pointing to general material available on the site rather than addressing individual users’ specific circumstances. Since its establishment in February 2002, GARD has answered over 22,000 requests about 6,000 rare and genetic diseases, as reported by the NIH.

### 4.3 Orphanet

Orphanet is a European platform dedicated to rare diseases and orphan drugs, led by the Institut National de la Santé et de la Recherche Médicale (INSERM) in collaboration with various countries and organizations, primarily within the European Union. The main objective of Orphanet is to provide high-quality information about rare diseases and ensure that all stakeholders have equitable access to knowledge. The platform also publishes a series of widely downloaded publications that present aggregated data on topics relevant to all rare diseases.

The inventory of rare diseases on Orphanet can be searched using disease names, gene names, symbols, or the disease’s “functional consequences” (disabilities), as well as other identifying numbers like the Online Mendelian Inheritance in Man (OMIM) number. A beta tool called PhenomizerOrphanet is also available to assist in clinical differential diagnosis through controlled vocabulary searches. Orphanet offers an “Encyclopedia for Patients,” an “Encyclopaedia for Professionals,” and “Emergency Guidelines” for healthcare professionals. However, it should be noted that the quantity of diseases addressed in the articles within the Encyclopedias. Is generally limited. The site’s content is accessible in multiple European languages and includes information on 6,172 diseases and 5,835 genes (Orphanet, 2021).

As stated on the website, all disease entries are written by specialists and undergo evaluation by peers. However, it’s important to acknowledge that the mentioned therapies may not be evidence-based due to the limited number of cases available for gathering evidence for or against a particular treatment.

### 4.4 Online Mendelian Inheritance in Man (OMIM)

Online Mendelian Inheritance in Man (OMIM) is an authoritative and freely accessible database containing comprehensive information about human genes and genetic traits, which is updated on a daily basis. The comprehensive summaries in OMIM include information about all identified Mendelian diseases and over 16,000 genes. The database focuses on establishing the connection between phenotype and genotype, and its articles are regularly updated, providing numerous links to additional genetics resources.

In the early 1960s, Dr. Victor A. McKusick launched the database known as Mendelian Inheritance in Man (MIM), originally intended as a catalog of Mendelian traits and disorders. This catalog was published in twelve book versions from 1966 to 1998. Subsequently, in 1985, an online version called OMIM was developed through a collaboration between the National Library of Medicine and the William H. Welch Medical Library at Johns Hopkins. It became widely available on the Internet in 1987. Subsequently, in 1995, the National Center for Biotechnology Information (NCBI) created the World Wide Web version of OMIM. Dr. Ada Hamosh leads the McKusick-Nathans Institute of Genetic Medicine at Johns Hopkins University School of Medicine, where OMIM is authored and edited (OMIM, 2021).

Unlike primary data databases, OMIM aggregates and summarizes essential information derived from expert reviews of the biomedical literature. Consequently, OMIM has played a pioneering role in naming and classifying genetic phenotypes (Amberger et al., 2015). A simple search in the OMIM database reveals numerous genes associated with various diseases, some of which exhibit multiple inheritance patterns.

### 4.5 LORIS MyeliNeuroGene rare disease database

In 2021, Spahr et al. (2021) introduced the LORIS MyeliNeuroGene rare disease database for conducting natural history studies and preparing for clinical trials. This online database for rare disease and needs subscription, it is not free access like OMIM or orphanet or GARD. Employing FDA-compliant databases for developing clinical trials with historical control data could significantly impact patients and families.

Spahr et al. (2021) created an accessible multi-modal database accessible via a web browser, which included genetics, imaging, behavioral, and patient-reported outcomes. The main goals were to increase the size of cohorts, identify surrogate markers, and foster international collaborations. The database contained a comprehensive range of information, such as family, perinatal, and developmental history, clinical examinations, diagnostic investigations, neurological evaluations (e.g., spasticity, dystonia, ataxia, etc.), disability measures, parental stress, and quality of life data.

Spahr et al. (2021) highlighted that their manuscript is the first to outline the requirements for adhering to Title 21 Code of Federal Regulations Part 11 Compliance. Subsequent studies will employ the tools developed in this project to characterize the natural progression of diverse rare diseases, with the goal of providing valuable insights to clinicians and researchers globally.

In summary, the choice of resource depends on specific research needs and interests. Each of these databases serves a unique purpose. NORD and GARD are more patient-focused, while Orphanet provides comprehensive European coverage. OMIM offers specialized genetic information for professionals, and LORIS MyeliNeuroGene is niche-focused on neurological diseases.

## 5 Conclusion and future perspectives

Genetic testing is becoming increasingly popular and accessible for both individuals and clinicians in today’s world. While challenges and obstacles persist, NGS technologies hold significant promise as the initial stage in genetic testing for rare disease diagnoses.

This paper focuses solely on certain aspects of NGS-based genetic testing in clinical implementation and omits other vital factors. These include genetic counseling to improve the patient-physician relationship, addressing ethnic considerations in the adoption and delivery of genetic testing, and educational initiatives aimed at promoting the acceptance of genetic testing in clinical settings.

The challenge of data interpretation remains a significant obstacle when employing routine clinical NGS for diagnosis. Dealing with large datasets and interpreting them requires substantial resources and expertise from bioinformaticians. These datasets contain information on variations that need to be classified for accurate diagnosis. Although AI shows great potential in healthcare, it faces challenges, including the increasing data volume and associated costs from automated computing. AI systems demand specialized computational resources for swift data processing, making them expensive. Additionally, AI-based solutions require proper training and understanding by intended users before being integrated into routine clinical practice. Addressing ethical concerns regarding patient data use is critical, necessitating ethical standards and procedures to ensure patient safety and privacy.

AI is beginning to tap into its potential to enhance clinical usefulness and diagnostic capabilities by supplementing phenome-wide and genome-wide data profiles. iBoth government agencies and professional communities are actively supporting and initiating efforts to standardize regulations for NGS-based testing and AI applications. When dealing with rare diseases, further research is needed as traditional monogenic models may not be sufficient. Exploring the digenic/oligogenic model and investigating polygenic causes for undiagnosed cases could provide valuable insights (Katsanis et al., 2001; Hoefele et al., 2007; Boisson-Dupuis et al., 2018; Posey, 2019).
